# *Clonorchis sinensis* infection are associated with *calcium phosphate* gallbladder stones: A single-center retrospective observational study in China

**DOI:** 10.1097/MD.0000000000045739

**Published:** 2025-11-14

**Authors:** Rui-Hong Ma, Xiao-Bing Luo, Yu Peng Liu, Qin Li

**Affiliations:** a Department of Clinical Laboratory, Guangzhou Nansha People’s Hospital, Guangzhou, Guangdong, China; b Yale Cancer Center, Yale School of Medicine, New Haven, CT.

**Keywords:** calcium phosphate, *Clonorchis sinensis*, Fourier transform infrared spectroscopy, gallbladder stones, scanning electron microscopy

## Abstract

Limited studies have been conducted on calcium phosphate gallbladder stones, to enhance our comprehension of the formation, we investigated the clinical characteristics and the potential link between calcium phosphate gallbladder stones and *Clonorchis sinensis (C sinensis*) infection. A retrospective observational study was conducted involving 4451 patients who underwent gallbladder-preserving cholelithotomy between January 2013 and January 2022. Based on the regional prevalence of *C sinensis* infection, the patients were categorized into 2 groups: those from high-incidence regions and those from low-incidence regions. We analyzed their appearance, texture, and clinical attributes. Furthermore, we utilized light microscopy to detect *C sinensis* eggs and χ^2^ tests to analyze the detection rate. Additionally, Fourier transform infrared spectroscopy was employed to analyze stone composition, and scanning electron microscopy combined with element distribution analysis (SEM-EDAX) was used to observe microstructure and element distribution. A total of 245 cases were identified as calcium phosphate stones, which constituted 5.50% (245/4451) of all gallbladder stone cases. These stones exhibited various degrees of bilirubinate mixing, with nearly 19.18% (47/245) being combined with calcium carbonate (CaCO3). The presence of *C sinensis* eggs in calcium phosphate stones was significantly higher compared to non-calcium phosphate–CaCO_3_–bilirubinate stones (*P* < .05). Moreover, the prevalence of calcium phosphate stones was notably greater in *C sinensis* high-incidence regions than in low-incidence regions (*P* < .05). Calcium phosphate stones represent a significant subset of gallbladder stones. They frequently occur in combination with bilirubinate in varying proportions, and less commonly with other components. The development of calcium phosphate stones was associated with *C sinensis* infection. Therefore, effective prevention of this parasitic infection is essential for reducing the risk of calcium phosphate stone formation.

## 1. Introduction

Cholecystolithiasis, commonly known as gallbladder stones, is characterized by the formation of calculi within the gallbladder and represents one of the most prevalent and economically significant gastrointestinal disorders worldwide.^[1–3]^ Influenced by hereditary, environmental, and lifestyle factors, gallstone disease has been increasing in global prevalence and is expected to continue rising.^[4–7]^ A recent nationwide study in China reported an age- and gender-standardized prevalence of 5.13%, with gallbladder stones accounting for 76.3% of all cases.^[8]^ Despite its significance, the etiology and pathogenesis of gallstones remain incompletely understood, with an absence of comprehensive prevention strategies and efficacious nonsurgical therapies. Unraveling the chemical constituents of gallstones is pivotal in deciphering their mechanisms of formation.^[9–11]^ Numerous methodologies exist for stone analysis, encompassing chemical, physical, and microstructural approaches. Each method offers distinct advantages. While chemical analysis stands as a traditional and widely employed technique due to its speed, simplicity, and cost-efficiency, its limitations include high sample consumption and susceptibility to inaccuracy. Fourier transform infrared (FTIR) spectroscopy, a technique based on the absorption spectra of molecular components resulting from atomic vibrations, offers a rapid, cost-effective, and precise means of gallstone analysis. Notably, it is applicable across all gallstone types and requires only minimal sample quantities, obviating the need for pretreatment.^[12,13]^ Scanning electron microscopy (SEM) facilitates microscopic morphology analysis, while its combination with element distribution analysis (SEM-EDAX) offers insight into the microstructure and element composition of substances. Presently, cholesterol and calcium bilirubinate emerge as the primary chemical constituents in gallstones, influenced by a range of causative factors that promote their precipitation in bile.^[14–17]^ Nevertheless, calcium phosphate, traditionally considered a minor element in gallstones compared to its prevalence in urinary calculi, has been undergoing a reevaluation.^[18]^ Recent investigations on 102 gallstones extracted from Sri Lankan patients unveiled that 25% of these stones featured calcium phosphate alongside calcium bilirubinate and calcium carbonate (CaCO_3_), particularly in pigment stones.^[19]^ Furthermore, experimental studies utilizing model biles have indicated that calcium phosphate precipitates possess a heightened ability to stimulate cholesterol crystallization, surpassing even the impact of soluble calcium.^[20]^

Building upon our previous retrospective observational study, which systematically classified 807 gallstone samples from the Department of General Surgery of People’s Hospital of Nansha, Guangzhou, between January 2009 and November 2012. The patient cohort comprised 423 males (aged 17–77, mean = 45.39 ± 11.87 years) and 384 females (aged 10–80, mean = 46.07 ± 12.89 years). Our initial findings indicated that gallstones predominantly composed of calcium phosphate represented a modest proportion, accounting for only 1.5% of cases.^[21]^ Intriguingly, as the sample size expanded, our current investigation in the present study underscored a noteworthy shift. Calcium phosphate stones, encompassing both those predominantly composed of calcium phosphate and those featuring calcium phosphate in combination with other constituents, now represent a significantly larger portion at 5.5%, suggesting potential regional disparities. To delve into the intricate formation of calcium phosphate gallbladder stones, we embarked on a meticulous study centered around 245 such stones.

Clonorchiasis, commonly referred to as Chinese liver fluke disease, is a significant amphixenosis prevalent primarily in East and Southeast Asia.^[22,23]^ It is well-established that cholecystolithiasis is frequently associated with Clonorchis sinensis (*C sinensis*) infection. Several studies have indicated a strong correlation between this parasitic infection and the formation of intrahepatic bile duct stones.^[24,25]^ Furthermore, our previous research confirmed that *C sinensis* infection is a major risk factor for the development of gallbladder stones, particularly those composed of CaCO_3_.^[26]^ Nevertheless, no studies have been conducted on the relationship between calcium phosphate stones and *C sinensis* infection. We comprehensively analyzed the external characteristics, internal texture, and the detection of *C sinensis* eggs, and dissected the components and microstructure of these stones in gallbladder from 245 patients to unravel their underlying mechanisms of development. The findings of this study represent important contributions to the prevention and management of calcium phosphate stones.

## 2. Materials and methods

### 2.1. Ethics statement

The protocols for sample collection were comprehensively communicated to all participating patients. Prior to inclusion, each patient provided informed consent, and for minors/children involved, written informed consent was obtained from their guardians. The research strictly adhered to the principles outlined in the Declaration of Helsinki of 1975, as revised in 1983 and 1989. Approval for the study was granted by the Ethics Committee of the Sixth People’s Hospital of Nansha, Guangzhou.

### 2.2. Study inclusion and exclusion criteria

Inclusion criteria: patients met the operation criteria and accepted gallbladder-preserving cholelithotomies in the Department of General Surgery at People’s Hospital of Nansha between January 2013 and January 2022. Exclusion criteria included patients with common bile duct stones and/or jaundice, acute cholecystitis, gallbladder carcinoma, or lack of a gallbladder cavity.

### 2.3. Composition analysis of gallbladder stones by Fourier transform infrared (FTIR) spectroscopy

The main components were analyzed using a Bruker (TENSOR27, Germany) FTIR spectrometer. A 2 mg sample of each layer was weighed if the layered structures were distinct. In amorphous stones, 2 mg samples were weighed directly. The samples were mixed with KBr at a ratio of 1:150, ground thoroughly, and used to make discs. The main components were analyzed using a Bruker (TENSOR27, Germany) FTIR spectrometer in the frequency range of 400 to 4000 cm^−1^, at 4 cm^−1^ resolution. Control substances (99% pure standard) were obtained from Sigma Chemical Company (St. Louis, MO). The composition of gallstones was ascertained by comparing them to standard control spectra.^[27,28]^

### 2.4. Definition of groups

Based on stone components, they were divided into 3 groups included calcium phosphate stones group, CaCO_3_ and/or bilirubinate stones group, as well as non-calcium phosphate–CaCO_3_–bilirubinate stones groups (including cholesterol stone, calcium stearate, and cholesterol–bilirubinate mixed stone, etc).

Based on epidemiological data of *C sinensis* infection, gallbladder stone cases are classified according to the patients’ geographic origins into high-incidence and low-incidence regions for this parasitic infection. High-incidence areas primarily include Guangdong and Guangxi provinces, whereas low-incidence regions encompass areas such as Shanxi, Gansu, and Xinjiang.^[29]^

### 2.5. Microscopic examination of ground gallbladder stone

Gallbladder stones collected during surgical procedures were rinsed twice with distilled water and air-dried at 40°C overnight. Subsequently, the stones were split using a surgical knife. The layers were examined macroscopically, in cases where distinct layered structures were present, a 10 mg stone sample from each layer was weighed. For amorphous stones, 10 mg was directly weighed. The stone sample was then mixed with 300 μL of normal saline, thoroughly ground in a mortar, and filtered using a 260-mesh nylon yarn. Filtrates from each stone sample were applied onto 3 to 4 slides, appropriately labeled, and evaluated for the presence or absence of *C sinensis* eggs using an Olympus System Microscope (BX51, Japan). Infection with *C sinensis* was defined by the detection of its eggs within the gallbladder stones. The detection rate was subsequently calculated within each group.

### 2.6. Microstructure and element analysis with SEM-EDAX

A selection of stones from calcium phosphate stones group, CaCO_3_ and/or bilirubinate stones group, as well as non-calcium phosphate–CaCO_3_–bilirubinate stones groups was randomly chosen for SEM-EDAX analysis. These stones were affixed to a sample table using an electroconductive adhesive and dried at 40°C overnight. Subsequently, the dried samples underwent gold sputter-coating (ETD-2000, Beijing Elaborate Technology Development, China) and were observed using a ZEISS SEM (EVO LS10, Cambridge, Germany) before being photographed. Element composition and distribution were analyzed using an X-ray energy spectrometer (X-Max, OXFORD, England), following a procedure elucidated in our prior work.^[21]^

### 2.7. Data collection

Demographics and patient origin regions were collected from their medical records, *C sinensis eggs* detection, composition and microstructure of the stones were collected from our data.

### 2.8. Statistical analysis

The experimental data were analyzed using *SPSS* v.16.0 software. Chi-square tests were employed to analyze the detection rate, with statistical significance set at *P* < .05. For multiple comparisons, a contingency table χ^2^ test was used, and the re-inspection standard was α′ = α/*N (N* = *n*(*n* − 1)/2 + 1), with *n* representing the number of groups participating in the test, including calcium phosphate stones group, CaCO_3_ and/or bilirubinate stones group, as well as non-calcium phosphate–CaCO_3_–bilirubinate stones groups, α denotes the alpha level, and α′ denotes the corrected alpha.

## 3. Results

### 3.1. Participant characteristics

As shown in Table 1, a total of 4451 patients with gallbladder stones were sourced, among these, 245 patients exhibited calcium phosphate stones, comprising 142 males and 103 females, with ages ranging from 9 to 75 years (mean age 50.99 ± 12.19 years). Additionally, 2529 patients exhibited CaCO_3_ and/or bilirubinate stones, comprising 1521 males and 1008 females, with ages ranging from 8 to 83 years (mean age 50.28 ± 12.03 years). Besides, 1677 patients with non-calcium phosphate–CaCO_3_–bilirubinate stones were included, comprising 736 males and 941 females, with ages ranging from 12 to 79 years (mean age 43.59 ± 12.58 years). No significant gender ratio or age differences were observed between patients with calcium phosphate stones and those with bilirubinate and/or CaCO_3_ stones (*P* > .05). Nevertheless, a marked difference was noted when comparing the gender ratio and age of patients with calcium phosphate stones to those with non-calcium phosphate-bilirubinate- CaCO_3_ stones, as determined by an independent sample *t*-test (*P* < .05).

**Table 1:** Participant characteristics.

Stone types	Male	Female	Total	Average age (yr)
Calcium phosphate stone	142	103	245	50.99 ± 12.19
calcium carbonate and/or bilirubinate stone	1521	1008	2529	50.28 ± 12.03
Non-calcium phosphate–calcium carbonate–bilirubinate stone	736	941	1677	43.59 ± 12.58
Total	2399	2052	4451	47.80 ± 12.67

### 3.2. Appearance and FTIR analysis of calcium phosphate stones

**Figure 1.**
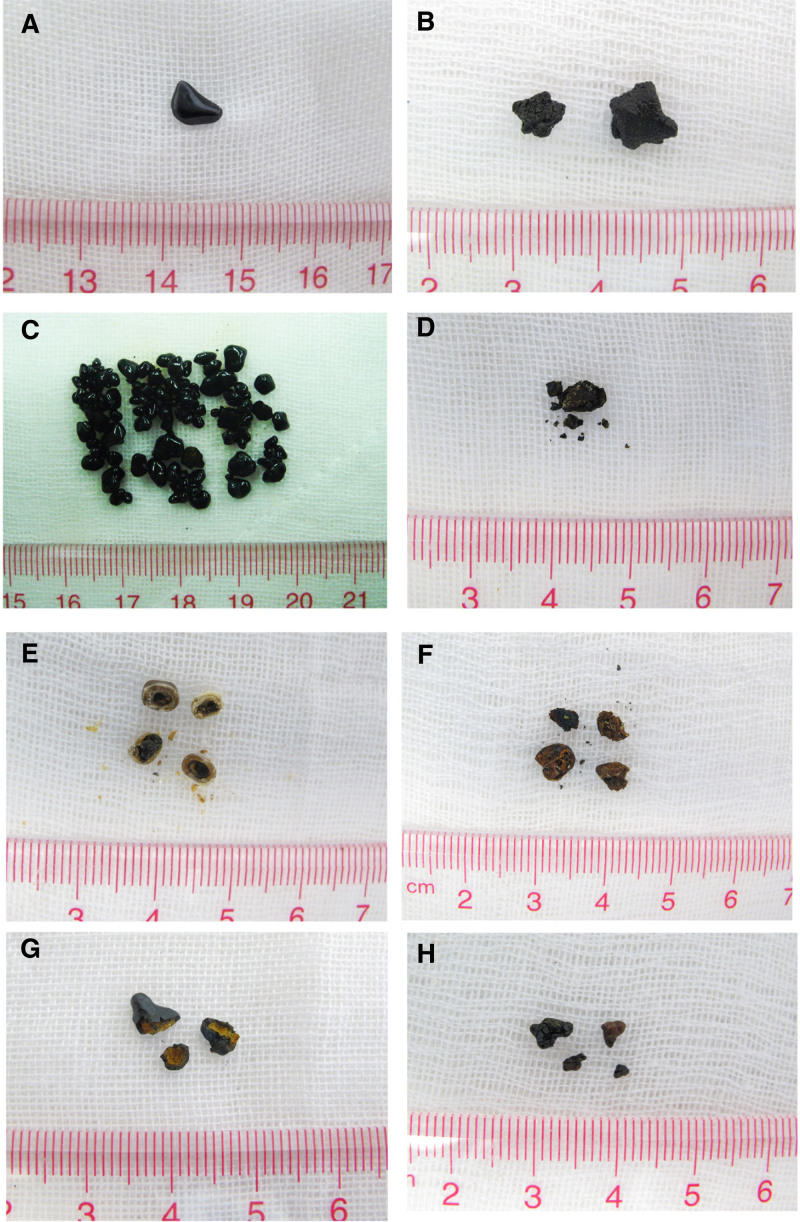
The appearance of calcium phosphate stones. (A, B) Calcium phosphate stones; (C, D) Bilirubinate–calcium phosphate mixed stone; (E) Cholesterol–calcium phosphate mixed stone; (F) Bilirubinate–calcium carbonate–calcium phosphate mixed stone; (G) Bilirubinate–calcium stearate–calcium phosphate mixed stone; (H) Cholesterol–bilirubinate–calcium phosphate mixed stone.

**Figure 2.**
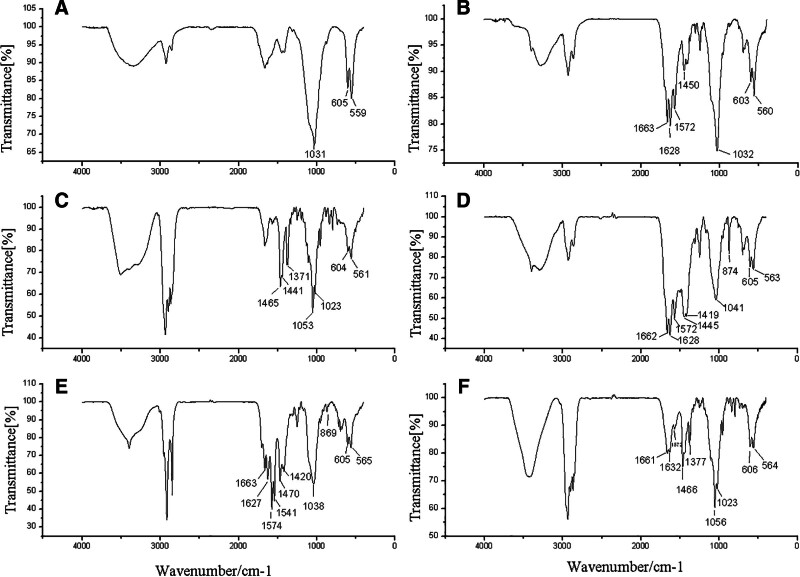
Typical IR spectrogram of calcium phosphate stones. (A) Calcium phosphate stones; (B) Bilirubinate–calcium phosphate mixed stone; (C) Cholesterol–calcium phosphate mixed stone; (D) Bilirubinate–calcium carbonate–calcium phosphate mixed stone; (E) Bilirubinate–calcium stearate–calcium phosphate mixed stone; (F) Cholesterol–bilirubinate–calcium phosphate mixed stone.

The external appearance of calcium phosphate stones was characterized by a black, irregular, cinder-like texture, with either smooth or rough surfaces and no discernible layering (Fig. 1A and B). Bilirubinate–calcium phosphate mixed stones showed a similar appearance but were accompanied by small white particles on the profile (Fig. 1C and D). Cholesterol–calcium phosphate mixed stones exhibited a brownish-yellow or celadon color and displayed a distinct layered structure in cross-section, with a soft outer layer and a brittle, gray-to-black core (Fig. 1E). Irregular bilirubinate–CaCO_3_–calcium phosphate mixed stones appeared black or brownish-black, and lacked a layered structure (Fig. 1F). In contrast, irregular bilirubinate–calcium phosphate–calcium stearate mixed stones had rough surfaces, some with layered profiles featuring an outer black layer and an umber or black mixed umber core (Fig. 1G). The irregular cholesterol–bilirubinate–calcium phosphate mixed stones were celadon in color with a rough surface, and some had layered profiles with a celadon outer layer and a gray or black core interspersed with white particles (Fig. 1H). Infrared spectroscopy of calcium phosphate stones showed a strong absorption peak for calcium phosphate and a weak peak for bilirubinate (Fig. 2A). Bilirubinate–calcium phosphate mixed stones exhibited strong absorption peaks for both compounds (Fig. 2B). Cholesterol–calcium phosphate mixed stones displayed prominent peaks corresponding to cholesterol and calcium phosphate (Fig. 2C). For bilirubinate–CaCO_3_–calcium phosphate mixed stones, strong absorption peaks were observed for bilirubinate, CaCO_3_, and calcium phosphate (Fig. 2D). Similarly, bilirubinate-calcium phosphate-calcium stearate mixed stones showed strong absorption peaks for bilirubinate, calcium phosphate, and calcium stearate (Fig. 2E). Finally, cholesterol–bilirubinate–calcium phosphate mixed stones exhibited strong absorption peaks for cholesterol, bilirubinate, and calcium phosphate (Fig. 2F).

### 3.3. Microstructure of calcium phosphate stones with different compositions under optical microscope

**Figure 3.**
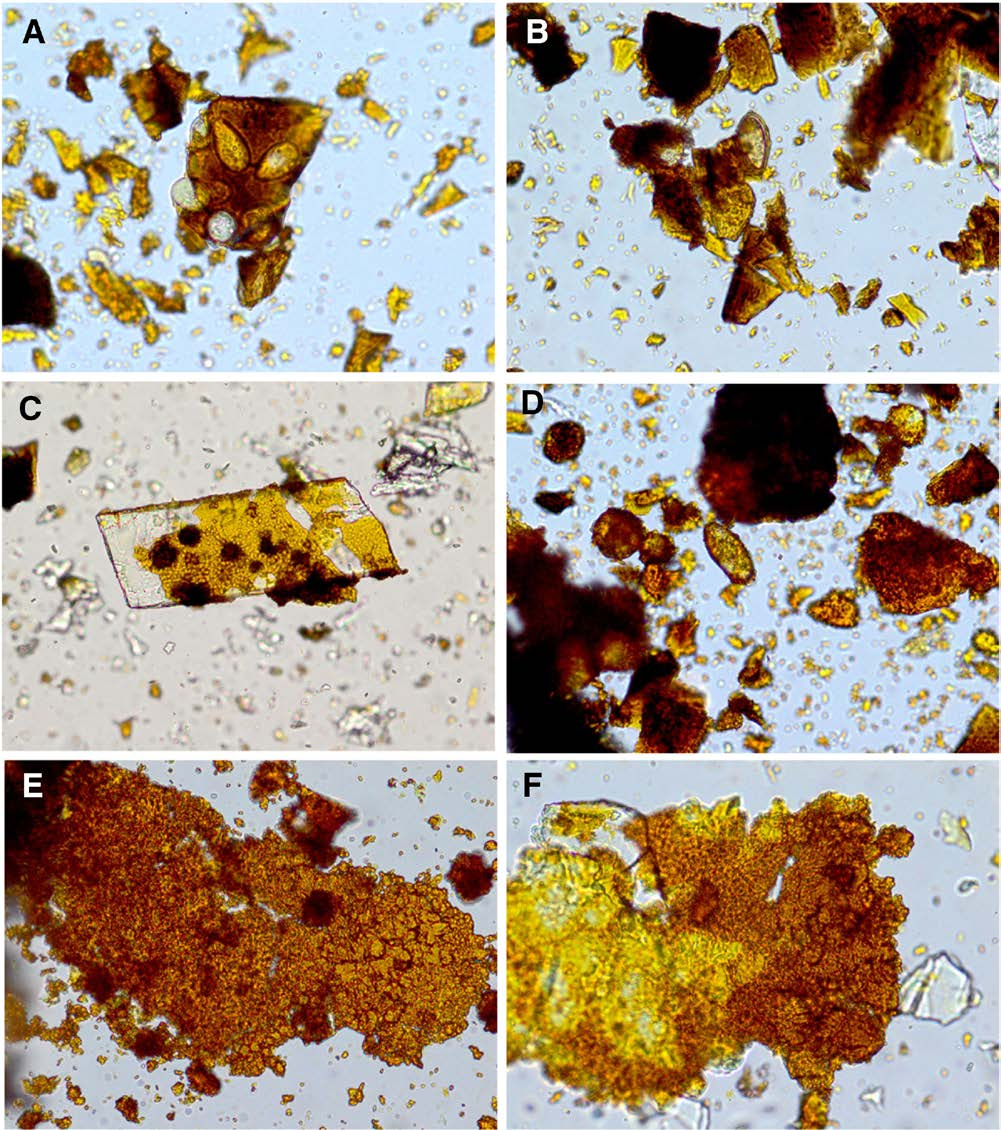
The microstructure of calcium phosphate crystals under optical microscope. (A, B) Bilirubinate–calcium phosphate mixed crystals; (C) Cholesterol–calcium phosphate mixed crystals; (D) Bilirubinate–calcium carbonate–calcium phosphate mixed crystals; (E) Bilirubinate–calcium stearate–calcium phosphate mixed crystals; (F) Cholesterol–bilirubinate–calcium phosphate mixed crystals.

In calcium phosphate and bilirubinate–calcium phosphate mixed stones observed under a microscope, yellow–orange peel-like calcium phosphate crystals were adhered to bilirubinate granules (Fig. 3A and B). *C sinensis* eggs could be detected in all calcium phosphate stones or calcium phosphate mixed stones, and they were mostly adhered to or enclosed by bilirubinate granules and/or calcium phosphate crystals as well as CaCO_3_ crystals (Fig. 3A, B, and D). Cholesterol–calcium phosphate mixed stones showcased cholesterol crystals wrapped in calcium phosphate crystals, often combined with bilirubinate granules (Fig. 3C). Irregular bilirubinate-CaCO_3_–calcium phosphate mixed stones exhibited adherence between bilirubinate granules, CaCO_3_ crystals, and calcium phosphate crystals (Fig. 3D). A similar trend was observed in bilirubinate–calcium stearate–calcium phosphate mixed stones, where irregular bilirubinate granules, calcium stearate crystals with honeycomb structure, and bulbiform calcium phosphate crystals adhered together (Fig. 3E). Furthermore, cholesterol crystals, bilirubinate granules, and calcium phosphate crystals were present in cholesterol–bilirubinate–calcium phosphate mixed stones (Fig. 3F).

### 3.4. Microstructure and elemental composition of calcium phosphate stones with different compositions under SEM

**Figure 4.**
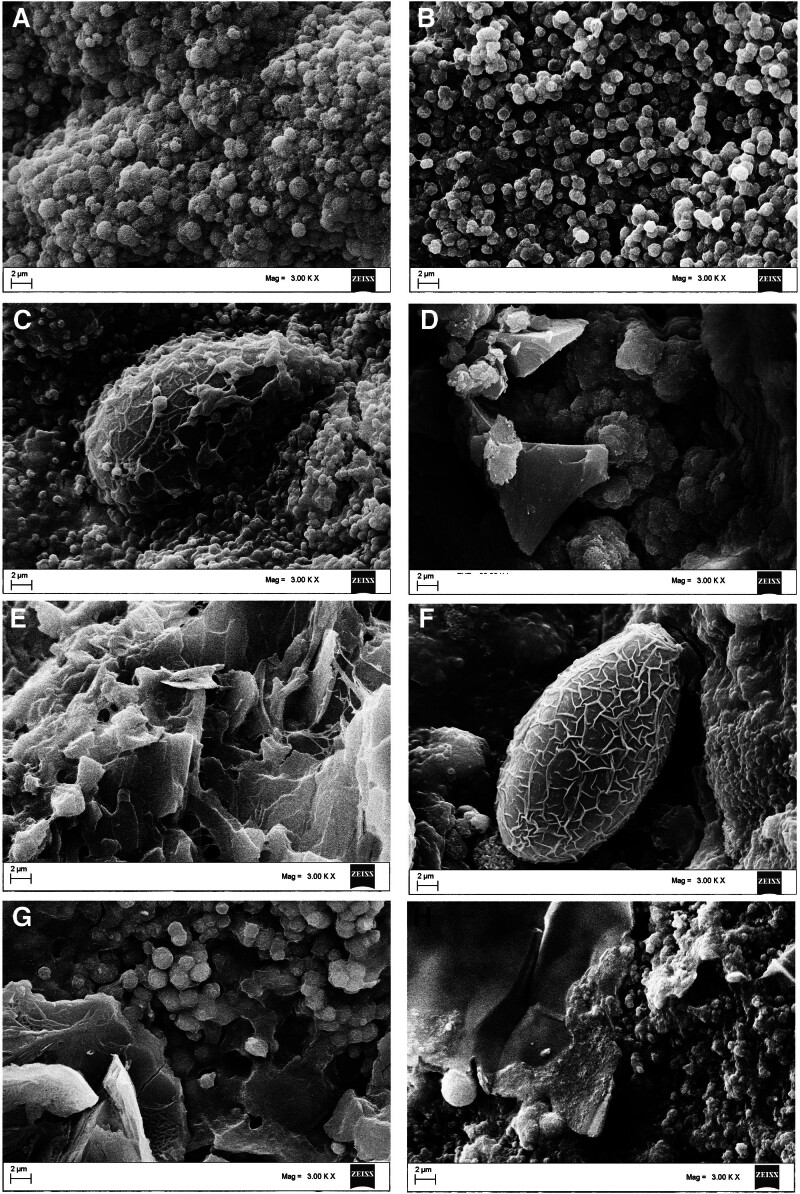
The microstructure of calcium phosphate crystals under SEM. (A, B) Calcium phosphate crystals; (C) *C sinensis* eggs and Calcium phosphate crystals; (D) Bilirubinate–calcium phosphate mixed crystals; (E) Cholesterol–calcium phosphate mixed crystals; (F) *C sinensis* eggs and bilirubinate–calcium carbonate–calcium phosphate mixed crystals; (G) Bilirubinate–calcium stearate–calcium phosphate mixed crystals; (H) Cholesterol–bilirubinate–calcium phosphate mixed crystals.

**Figure 5.**
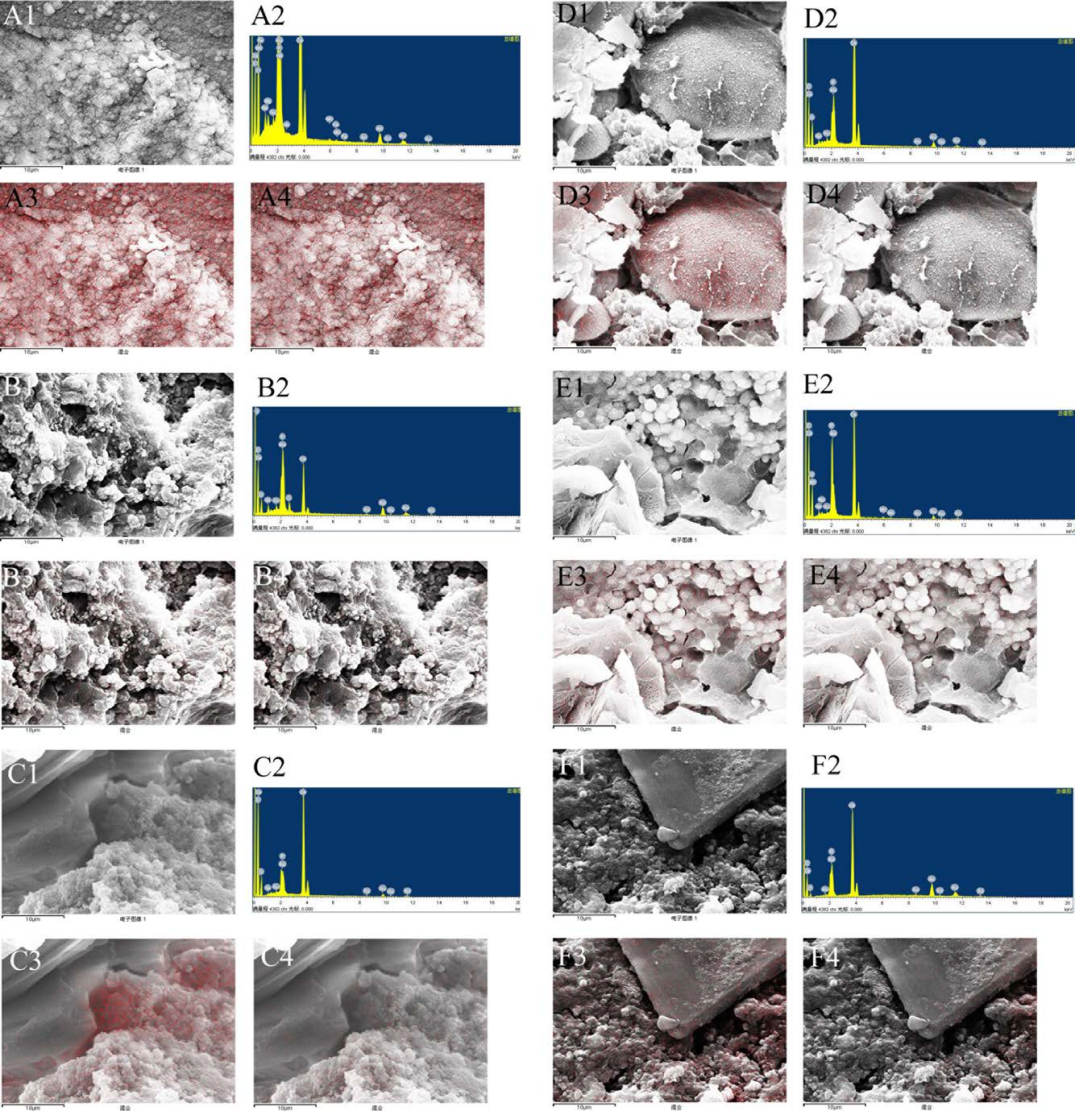
Elements distribution of calcium phosphate crystals and mixed crystals: (A1) Calcium phosphate crystals with a magnification of 3000 times, (A2) The energy spectrum of the micro field, (A3) calcium distribution, (A4) phosphorus distribution; (B1) Bilirubinate-calcium phosphate mixed crystals with a magnification of 3000 times, (B2) The energy spectrum of the micro field, (B3) calcium distribution, (B4) phosphorus distribution; (C1) Cholesterol–calcium phosphate mixed crystals with a magnification of 3000 times, (C2) The energy spectrum of the micro field, (C3) calcium distribution, (C4) phosphorus distribution; (D1) Bilirubinate–calcium carbonate–calcium phosphate mixed crystals with a magnification of 3000 times, (D2) The energy spectrum of the micro field, (D3) calcium distribution, (D4) phosphorus distribution; (E1) Bilirubinate–calcium stearate–calcium phosphate mixed crystals with a magnification of 3000 times, (E2) The energy spectrum of the micro field, (E3) calcium distribution, (E4) phosphorus distribution; (F1) Cholesterol–bilirubinate–calcium phosphate mixed crystals with a magnification of 3000 times, (F2) The energy spectrum of the micro field, (F3) calcium distribution, (F4) phosphorus distribution.

SEM analysis of calcium phosphate and bilirubinate–calcium phosphate mixed stones revealed echin-sphere or rough bulbiform or ceramisite-like calcium phosphate crystals adhering to irregular bilirubinate granules (Fig. 4A–D). *C sinensis* eggs could be detected in all calcium phosphate stones or calcium phosphate mixed stones, and mostly adhered to or were enveloped by bilirubinate granules, mucoid matter, and calcium phosphate crystals (Fig. 4C and F). Cholesterol–calcium phosphate mixed stones predominantly consisted of tightly stacked, plate-like or lamellar cholesterol crystals intertwined with orange peel-like calcium phosphate crystals (Fig. 4E). Irregular bilirubinate granules, bulbiform CaCO_3_ crystals, and orange peel-like calcium phosphate crystals constituted the bulk of bilirubinate–CaCO_3_–calcium phosphate mixed stones (Fig. 4F). Irregular bilirubinate granules, calcium stearate crystals with honeycomb structures, and bulbiform calcium phosphate crystals characterized bilirubinate–calcium stearate–calcium phosphate mixed stones (Fig. 4G). Cholesterol–bilirubinate–calcium phosphate mixed stones consisted mainly of plate-like cholesterol crystals, irregular bilirubinate granules, and echin-sphere calcium phosphate crystals (Fig. 4H). The elemental distribution of calcium phosphate stones and various calcium phosphate-containing mixed stones is shown in Figure 5. Calcium phosphate stones exhibited high levels of both calcium and phosphorus distributed in calcium phosphate crystals (Fig. 5A). Bilirubinate–calcium phosphate mixed stones also showed high content of calcium and phosphorus primarily distributed in calcium phosphate crystals, though lower than that in pure calcium phosphate stones (Fig. 5B). Cholesterol–calcium phosphate mixed stones displayed high calcium but relatively low phosphorus content distributed mainly in calcium phosphate crystals (Fig. 5C). Bilirubinate–CaCO_3_–calcium phosphate mixed stones had comparable calcium levels but reduced phosphorus compared to pure calcium phosphate stones, calcium was mainly distributed in CaCO_3_ and calcium phosphate crystals, while phosphorus mainly distributed in calcium phosphate crystals (Fig. 5D). In contrast, bilirubinate–calcium stearate–calcium phosphate mixed stones contained relatively low amounts of both elements distributed mainly in calcium phosphate crystals (Fig. 5E). Cholesterol–bilirubinate–calcium phosphate mixed stones exhibited high calcium and low phosphorus content distributed mainly in calcium phosphate crystals (Fig. 5F). The characteristics of the different types of calcium phosphate stones have been summarized in Table 2.

**Table 2:** Characteristics of the different types of calcium phosphate stones.

Stone types	Appearance	FTIR analysis	Microstructure under optical microscope	Microstructure under SEM	Elemental composition
Pure calcium phosphate stone	Black, irregular, cinder-like texture, either smooth or rough surfaces.	A strong absorption peak for calcium phosphate and a weak peak for bilirubinate.	Yellow–orange peel-like calcium phosphate crystals adhering to a small amount of bilirubinate granules.	Echin-sphere or rough bulbiform or ceramisite-like calcium phosphate crystals adhering to a small amount of irregular bilirubinate granules.	High levels of both calcium and phosphorus distributed in calcium phosphate crystals.
Bilirubinate–calcium phosphate mixed stone	A similar appearance but with small white particles on the profile.	Strong absorption peaks for both compounds.	Yellow–orange peel-like calcium phosphate crystals adhering to bilirubinate granules.	Echin-sphere or rough bulbiform or ceramisite-like calcium phosphate crystals adhering to irregular bilirubinate granules.	High content of calcium and phosphorus primarily distributed in calcium phosphate crystals, though lower than that in pure calcium phosphate stones.
Cholesterol–calcium phosphate mixed stone	Brownish–yellow or celadon color, a distinct layered structure in cross-section, with a soft outer layer and a brittle, gray-to-black core.	Prominent peaks corresponding to cholesterol and calcium phosphate.	Cholesterol crystals wrapped in calcium phosphate crystals, often combined with bilirubinate granules.	Tightly stacked, plate-like or lamellar cholesterol crystals intertwined with orange peel-like calcium phosphate crystals.	High calcium but relatively low phosphorus content distributed mainly in calcium phosphate crystals.
Bilirubinate-calcium carbonate–calcium phosphate mixed stone	Black or brownish-black, and lacked a layered structure.	Strong absorption peaks for bilirubinate, calcium carbonate, and calcium phosphate.	Adherence between bilirubinate granules, calcium carbonate crystals, and calcium phosphate crystals.	Irregular bilirubinate granules, bulbiform calcium carbonate crystals, and orange peel-like calcium phosphate crystals.	A comparable calcium levels but reduced phosphorus compared to pure calcium phosphate stones, calcium was mainly distributed in calcium carbonate and calcium phosphate crystals, while phosphorus mainly distributed in calcium phosphate crystals.
Bilirubinate–calcium stearate–calcium phosphate mixed stone	Rough surfaces, with layered profiles featuring an outer black layer and an umber or black mixed umber core.	Strong absorption peaks for bilirubinate, calcium phosphate, and calcium stearate.	Irregular bilirubinate granules, calcium stearate crystals with honeycomb structure, and bulbiform calcium phosphate crystals adhered together.	Irregular bilirubinate granules, calcium stearate crystals with honeycomb structures, and bulbiform calcium phosphate crystals.	Relatively low amounts of both calcium and phosphorus distributed mainly in calcium phosphate crystals.
Cholesterol–bilirubinate–calcium phosphate mixed stone	Celadon color, rough surface, some had layered profiles with a celadon outer layer and a gray or black core interspersed with white particles.	Strong absorption peaks for cholesterol, bilirubinate, and calcium phosphate.	Cholesterol crystals, bilirubinate granules, and calcium phosphate crystals were present.	Plate-like cholesterol crystals, irregular bilirubinate granules, and echin-sphere calcium phosphate crystals.	High calcium and low phosphorus content distributed mainly in calcium phosphate crystals.

### 3.5. Detection of *C sinensis* eggs in calcium phosphate stones

The detection rate of *C sinensis* eggs in calcium phosphate stones was 51.4% (126/245). which was not significantly different from that in CaCO_3_ and/or bilirubinate stones (53.2%, 1346/2529). However, a substantial difference was noted when compared with non-calcium phosphate–CaCO_3_–bilirubinate stones, where the detection rate was 18.5% (311/1677) (*P* < .05). Furthermore, calcium phosphate stones accounted for 7.1% (126/1783) of egg-positive stones and 4.5% (119/2668) of egg-negative stones, a difference that was also statistically significant (*P* < .05). In addition, the proportion of calcium phosphate stones was higher in high-incidence regions of clonorchiasis (6.8%,224/3306) compared to low-incidence regions (1.8%, 21/1145). Additionally, the proportion of CaCO_3_ and/or bilirubinate stones was also notably higher in high-incidence regions (2245/3306 vs 304/1145), while the proportion of non-calcium phosphate–CaCO_3_–bilirubinate stones was lower in these regions (857/3306 vs 820/1145), as shown in Table 3.

**Table 3:** Distribution of *C sinensis* egg detection.

Stone types	Egg-positive	Egg-negative	Total	Clonorchiasis high-incidence region	Clonorchiasis low-incidence region	Total
Calcium phosphate stone	126	119	245	224	21	245
calcium carbonate and/or bilirubinate stone	1346	1183	2529	2225	304	2529
Non-calcium phosphate–calcium carbonate–bilirubinate stone	311	1366	1677	857	820	1677
Total	1783	2668	4451	3306	1145	4451

## 4. Discussion

Calcium phosphate, traditionally regarded as a minor component in gallstones, is currently undergoing reevaluation. In this study, a total of 4451 gallbladder stones were classified, among which 245 were identified as calcium phosphate stones, accounting for 5.5%. Demographic characteristics of patients with calcium phosphate stones were similar to those with CaCO_3_ and/or bilirubinate stones, but distinct from those with non-calcium phosphate–CaCO_3_–bilirubinate stones.^[26]^ These findings suggest that calcium phosphate stones may share etiological factors with CaCO_3_ and/or bilirubinate stones, but differ from non-calcium phosphate–CaCO_3_–bilirubinate stones.

Gallbladder calcium phosphate stones markedly differ from their urinary counterparts. While urinary calcium phosphate stones commonly appear as grayish-white or lotus root-colored with a brittle texture, often found in the kidney and ureter of female patients and closely related to urinary tract infection,^[30–32]^ or usually appeared in the core and mixed with calcium oxalate which appeared in the outer layer with an earthy gray appearance, gallbladder calcium phosphate stones manifest as black, irregular, cinder-like structures with variable surface smoothness. These gallbladder stones are typically devoid of a layered profile except for some bilirubinate–calcium phosphate–calcium stearate mixed stones, cholesterol–calcium phosphate mixed stones and cholesterol–bilirubinate–calcium phosphate mixed stones. Predominantly observed in male patients, these stones are usually mixed with bilirubinate to varying degrees, occasionally with the addition of CaCO_3_, and rarely with cholesterol or calcium stearate. The structure and layering patterns further indicate divergent formation factors among different stone constituents. Stones composed of calcium phosphate, bilirubin, and CaCO_3_ typically lack discernible layering. Microscopic examination under both light and SEM reveals a relatively compact internal arrangement, indicating that these stones may share similar formative factors. In contrast, when these components are combined with cholesterol or calcium stearate, distinct layered structures frequently develop. Microscopic analysis shows a looser, more porous architecture, suggesting the involvement of different formation mechanisms.

Intriguingly, the detection rate of *C sinensis* eggs is significantly higher in calcium phosphate stones and CaCO_3_ and/or bilirubinate, compared to non-calcium phosphate–CaCO_3_–bilirubinate stones. Furthermore, calcium phosphate, CaCO_3_ and/or bilirubinate stones possess elevated proportions in clonorchiasis high-incidence regions, in contrast to clonorchiasis low-incidence regions, while non-calcium phosphate–CaCO_3_–bilirubinate stones exhibit the opposite trend. This observation suggests a potential link between calcium phosphate stone formation and *C sinensis* infection, echoing findings associating *C sinensis* infection with bilirubinate and CaCO_3_ stones. This may stem from alterations in the bile microenvironment induced by *C sinensis* infection, facilitating the deposition of calcium salts like calcium phosphate, CaCO_3_ and calcium bilirubinate, ultimately fostering gallstone formation.^[26]^

The crystal types of urinary calcium phosphate stones primarily encompass hydroxyapatite or carbonate apatite, characterized by echin-sphere, tree bark-like, rough bulbiform, or ceramisite calcium phosphate crystals.^[30]^ In contrast, biliary calcium phosphate stones mainly comprise hydroxyapatite blended with carbonate apatite and amorphous calcium phosphate. The structural mechanism of urinary calcium phosphate stone formation suggests that bacterial infections, including nanobacterial infections, may trigger central calcium phosphate deposits, subsequently gathering other crystalline components around them.^[33,34]^ Despite similarities, limited research has explored gallbladder calcium phosphate stones in relation to bile bacteria or nanobacterial infections, warranting further investigation. We speculate that bacterial infection contributes to the development of calcium phosphate gallstones through multiple pathways. It induces an alkaline microenvironment that promotes the precipitation of calcium phosphate. Additionally, bacterial surfaces act as nucleation sites, facilitating crystal aggregation and stone growth. The associated infection and inflammatory response impair gallbladder motility, reducing emptying efficiency and resulting in bile stasis. This leads to concentrated bile with elevated levels of calcium and phosphorus, further enhancing the formation and growth of calcium phosphate gallstones.

Biomineralization of calcium phosphate entails several potential phases, including DCPD, OCP, HAP, and various ACPs.^[35]^ Our previous study on urinary stones highlighted the presence of brushite in urinary stones but not biliary stones, owing to the slightly acidic urine environment favoring brushite stability.^[36]^ In contrast, biliary stones, formed in the slightly alkaline bile environment, contained HAP and ACP. Additionally, studies on synthetic calcium phosphate concretions produced in simulated body fluid echoed the ultrafine structures of certain phosphate calculi.^[37]^ This resonance extends to the ultrafine structure of hydroxyapatite, revealing distinct spherical agglomerates and columnar crystals within structureless HAP phases, proposing mechanisms involving aggregate formation and retention within cavities.^[38]^ The semblance between these studies’ findings and the microstructure of calcium phosphate crystals in gallbladder stones underscores potential shared formation mechanisms. The possible mechanisms underlying the formation of gallbladder calcium phosphate stones in relation to *C sinensis* infection were as follows: *C sinensis* infection disrupts bile metabolism, resulting in an alkaline microenvironment that promotes the precipitation and stabilization of calcium phosphate crystals.^[23]^ Additionally, parasite eggs and deceased organisms serve as nucleation sites, facilitating crystal aggregation and stone encapsulation. Besides, the accompanying inflammatory response and impaired gallbladder contractility further exacerbate biliary stasis, creating conditions conducive to stone development and formation.

Conclusion: Calcium phosphate stones constitute a notable portion of gallbladder stones. These stones likely share common etiological factors with CaCO_3_ and/or bilirubinate stones, yet differ fundamentally from non-calcium phosphate–CaCO_3_–bilirubinate stone types. Their formation may be associated with both *C sinensis* infection and bacterial infection. Notably, the potential mechanisms through which *C sinensis* and bacterial infections contribute to calcium phosphate gallstone formation exhibit considerable similarity. Integrated strategies aimed at preventing biliary tract infections, particularly from *C sinensis*, represent a fundamental component of reducing the population-level incidence of calcium phosphate gallstones. Nevertheless, the molecular mechanisms involved in gallbladder stone formation associated with *C sinensis* infection require further investigation.

Study strengths: Methodological advantages: FTIR, SEM and X-ray energy spectrometer were combine used to identify the composition and microstructure of the stones precisely. Diverse sources of participants: a large number of participants were recruited from multiple provinces across China, ensuring a broad and diverse sample that enhances the representativeness of the results.

Limits: The sample sizes for some stones were relatively small, which may affect the accuracy of their compositional analysis. However, these cases accounted for a very low proportion of the total sample and are unlikely to influence the overall findings. Since bacterial infection in the biliary tract was not within the scope of this study, no specific data on this aspect are available; this will be addressed in future research.

## Acknowledgments

We are grateful to Yu-yang Feng, Xiao-feng Wang in the General Surgery Department of our hospital for kindly providing the specimens. We thank our colleagues in the General Surgery Department and the operating room for their enthusiastic help, and the leaders of our hospital for their support.

## Author contributions

**Conceptualization:** Rui-Hong Ma.

**Data curation:** Rui-Hong Ma.

**Formal analysis:** Xiao-Bing Luo.

**Investigation:** Yu Peng Liu.

**Methodology:** Xiao-Bing Luo.

**Resources:** Yu Peng Liu.

**Writing – original draft:** Rui-Hong Ma.

**Writing – review & editing:** Qin Li.
